# Achieving affordable critical care in low-income and middle-income countries

**DOI:** 10.1136/bmjgh-2019-001675

**Published:** 2019-06-19

**Authors:** Hugo C Turner, Nguyen Van Hao, Sophie Yacoub, Van Minh Tu Hoang, David A Clifton, Guy E Thwaites, Arjen M Dondorp, C Louise Thwaites, Nguyen Van Vinh Chau

**Affiliations:** 1 Oxford University Clinical Research Unit, Wellcome Africa Asia Programme, Ho Chi Minh City, Vietnam; 2 Nuffield Department of Medicine, Centre for Tropical Medicine and Global Health, University of Oxford, Oxford, UK; 3 Hospital for Tropical Diseases, Ho Chi Minh City, Vietnam; 4 Department of Engineering Science, Institute of Biomedical Engineering, University of Oxford, Oxford, UK; 5 Mahidol-Oxford Tropical Medicine Research Unit, Faculty of Tropical Medicine, Mahidol University, Bangkok, Thailand

**Keywords:** health economics, health systems, public health

**Summary box**

- Improving the quality and availability of critical care is essential for reducing the burden of preventable deaths in low-income and middle-income countries.
- The conventional high-income country model, based on resource-intensive intensive care units with expensive monitoring and supportive equipment and large numbers of highly trained staff, is unlikely to be suitable for these settings.
- Currently, costs severely restrict access to critical care in low-income and middle-income countries, and there is an urgent need to develop an alternative affordable critical care model for these settings.
- Innovative technology and digital health may offer part of the solution and enable the development of an affordable, sustainable and scalable model of critical care in resource-limited settings.

## Introduction

In 2016, an estimated 8.6 million premature deaths occurred in low-income and middle-income countries (LMICs) from causes that ‘should not occur in the presence of timely and effective healthcare’. Improving the quality and availability of critical illness care in LMICs is essential if this burden is to be reduced,^1 2^ and even more important over the coming years as populations age and the prevalence of comorbidities, such as cardiovascular disease and diabetes, increase.^1^

Currently, capacity for critical illness care in many LMICs^3–5^ is limited. In high-income countries, there are generally between 5 and 30 intensive care unit (ICU) beds per 100 000 people.^2 3^ The limited data available indicate that in LMICs, there are between 0.1 and 2.5 ICU beds per 100 000 people. Many countries are also transitioning from low to lower–middle income status, receiving less international healthcare aid^6^ which may limit resources available for expanding capacity. While, the expansion of private healthcare systems in LMICs may partly meet the increased demand, the quality of care delivered by such providers is variable and will be unaffordable for many.^2 7^

**Figure 1:**
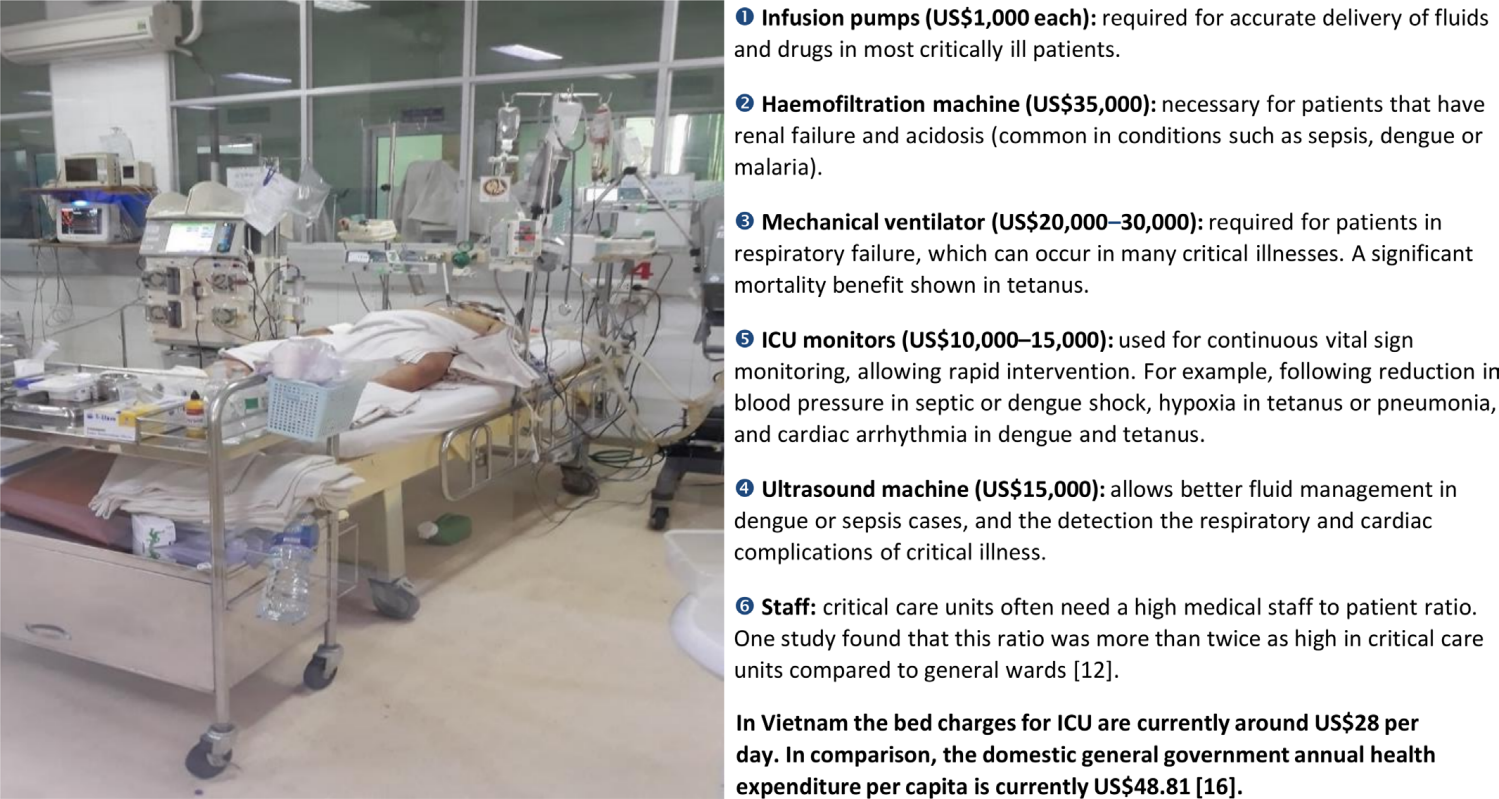
The costs for the monitoring and supportive equipment associated with an intensive care unit bed in Vietnam. Costs are based on quotes from commercial medical equipment distributors in Vietnam (2018 prices). Costs are not annualised.

Careful physiological monitoring is the cornerstone of good critical illness care.^8^ In the conventional high-income setting ICU model, monitoring is achieved with expensive equipment, high-quality laboratory support and large numbers of highly trained staff. In LMICs, this model is usually impractical as the required resources are either unavailable or too expensive.^4 5 9^ The figure 1 shows the predicted costs of providing a high-income country model ICU bed in Vietnam. Although this is just one case study, it highlights the magnitude of those costs. Counterintuitively, equipment costs can be substantially higher than for high-income countries, due to importation taxes and non-competitive pricing structures.

Maintaining operability of expensive ICU equipment is a further challenge in LMICs where there may be frequent power cuts and high ambient temperatures and humidity. Restricted availability of maintenance staff and replacement parts, means that equipment is often non-functioning or cannot be used to its full potential.^2 10^ Additionally, the paucity of appropriately trained staff and limited infection control measures can result in more frequent complications, which may worsen outcomes and further increase costs.^11 12^

Costing studies conducted in high-income countries have reported average costs of ICU care between US$1700 and 4500 per day (adjusted to 2014 prices).^13 14^ The delivery of critical care is less expensive in LMICs largely because of much lower labour costs; for example, a study based in an Indian hospital estimated the average daily cost of ICU care was US$109 (2014 prices).^15^ Although this amount may appear low, the average annual healthcare expenditure per capita across LMICs is only around 5% that of high-income countries.^16^

Furthermore, in LMICs, critical care costs are often not fully covered by the health/insurance systems and patients’ and their families can incur high out-of-pocket expenses.^17–19^ Currently costs severely restrict access to ICU care in LMICs, particularly for the socioeconomically disadvantaged and uninsured, and there is an urgent need to develop an alternative affordable critical care model for LMICs.^20^

## What can be done?

The emergence of new technologies, means there are huge opportunities to expand capacity and improve the care of critically ill patients in LMICs.^10 21 22^

A substantial proportion of critical care costs in LMICs are to cover staffing and fixed asset equipment costs as opposed to actual medications and laboratory tests.^15^ Methods impacting these may be a way of reducing costs, allowing expansion of capacity as well as improving the care quality.

Low-cost wearable devices offer a potentially affordable approach to physiological monitoring in LMICs, reducing the need for expensive commercial equipment and, combined with artificial intelligence (AI), may also improve care quality. Wearable devices, such as fitness trackers, have been used in ICU populations in high-income countries and have shown good correlation with conventionally-derived ECG data.^23^ AI and machine learning algorithms can be used to analyse ICU patients’ physiological data, learn from them and create computer-assisted decision-support systems.^22^ With simple modifications, low-cost wearables can feed data into AI systems which can then guide treatment decisions and diagnostics. A key advantage of this approach is that AI systems can compensate for the noisy, artefactual signals that typically arise from wearables. In high-income settings, AI algorithms have been shown to improve the management of sepsis and lower mortality.^24 25^ The ability of AI systems to continuously learn and adapt means that computer-assisted clinical decision-support systems can be tailored to the needs of a specific context or setting. Thus algorithms could be created to help in the management of diseases such as malaria, dengue and tetanus, which are uncommon in high-income ICU settings but are significant problems in LMICs. Importantly, existing libraries of analogous data sets acquired from western clinical settings can be used, along with smaller quantities of LMICs physiological data to permit ‘transfer learning’, in which complex predictive models can be retrained and recalibrated for use with low-cost sensors.

As well as physiological monitoring, point-of-care diagnostic and imaging devices are also increasingly available and affordable. Devices such as hand-held ultrasound probes connecting to a mobile phone could make equipping LMIC ICUs more feasible and cheaper, aiding diagnosis and management of patients. While a high degree of training is currently required to acquire and interpret images, future AI systems may provide operator guidance for inexperienced users and perform image interpretation, reducing requirements for highly trained staff and making the provision of critical care services outside of major urban hospitals significantly more feasible.

## What should happen next?

The potential for new technology to transform healthcare in LMICs is now widely accepted, but most innovations remain at the proof-of-concept stage or have only been tested in small pilot studies.^22 26^ The current challenge is bridging the gap between proof-of-concept and actual large-scale implementation.^22 27^

Moving forward, there is an urgent need to conduct implementation trials, to assess the actual effectiveness and feasibility of using these new digital technologies for critical care in LMICs. Successful innovation can only take place in close collaboration with end-user communities and a real understanding of the contextual need. In addition, the wide variety of critical care capacity and facilities within and between many LMICs means that new technologies should be designed to fit within the existing infrastructure. This will therefore require more than a simple design process but an active two-way partnership between all stakeholders and with considerations regarding scale-up taken into account from the start of the process.

The use of these new technologies also needs to be part of broader strategies to improve ICU performance. Other potential strategies for improving the delivery of critical care in LMICs include improving organisational structures, empowerment of nurses and locally generated clinical guidelines.^2 28^ In addition, in most LMICs, critical care is not currently a well-developed specialty. Consequently, the development of training and capacity building programmes is particularly important—not only for ICU physicians but also for nurses and other clinical personnel.^2^ It is vital that these training programmes, as well as covering specific ICU clinical skills also include basic management and organisational aspects of critical care.^2^ Successful initiatives such as Train-the-Trainer and peer-to-peer programmes have been shown to be successful in LMICs and could be further expanded.^29 30^

## Conclusion

Improving the quality and availability of critical care is essential for reducing the burden of preventable deaths in LMICs. There will be no one size fits all solution to this problem and a multifaceted approach is required. Nevertheless, greater utilisation of new technologies could be an important part of the solution. Through such innovation, critical care capacity could not only be increased but also be improved in quality and at a reduced cost. However, designing and implementing sustainable and scalable solutions is a significant challenge, requiring strong collaborations and real understanding between all stakeholders.
